# Supplementary material for: Are carbon emissions trading and green financial instruments synergistic? -Comprehensive quantitative research based on content analysis
Source: PLoS One. 2024 Mar 7;19(3):e0298601. doi: 10.1371/journal.pone.0298601 (PMC10919601; doi:10.1371/journal.pone.0298601)
Supplement: S1 Data — (ZIP) [file pone.0298601.s001.zip › Data/地级市Z_F工作报告.zip!/╘╞─╧/┴┘▓╫╩╨/└┤╫╘╣1⁄2╓┌║┼í╛┬φ┐╦╩2╛▌═°í┐_2014.docx]

各位代表：

　　现在，我代表市人民政府，向大会作政府工作报告，请予审议，并请市政协各位委员提出意见。

　　一、2013年和第二届市人民政府工作回顾

　　2013年是本届政府履职的最后一年。在省委、省政府和市委的坚强领导下，市人民政府认真贯彻落实党的十八大，十八届二中、三中全会，省委九届四次、五次全会，省十二届人大一次会议，市委三届五次全会和市二届人大七次会议精神，按照“稳中有进、稳中有好、稳中有快”的总要求，牢固树立“生态立市，绿色崛起”的发展理念，坚持“一产发展生态化、庄园化，二产发展绿色化、园区化，三产发展特色化、高端化”的三产联动发展思路，紧紧围绕“构筑生态高地，成就大美临沧”的发展目标，以“10大产业、10大园区、10大基础设施”建设为重点，着力打造“世界佤乡、天下茶仓，恒春之都、大美临沧”的城市品牌，抓项目、破瓶颈、彰特色、促跨越，全力打好县域经济、园区经济、民营经济三大战役。全市生产总值增长13.6%，规模以上固定资产投资增长37.6%，地方公共财政预算收入增长21.9%，社会消费品零售总额增长14.1%，外贸进出口总额增长64.6%，城镇居民人均可支配收入增长13.2%，农村居民人均纯收入增长17.6%，人口自然增长率为6.47‰，城镇登记失业率为3.57%，居民消费价格总水平涨幅2.4%，单位生产总值能耗下降1.02%。全市呈现经济较快发展、民族团结进步、民生持续改善、生态环境优良、边境和平安宁、社会和谐稳定的良好局面。

　　（一）农业产业结构进一步优化。坚持巩固农业基础地位不动摇，推进一产发展生态化、庄园化。全力抗击冬春干旱、夏秋洪涝、低温霜冻等自然灾害，粮食产量达98.6万吨，烤烟收购总量达110.2万担，冬季农业开发完成总产值16.5亿元，增长24.1%。以高原特色农业示范县、农业产业园和农业庄园建设为重点，带动高原特色农业加快发展，不断优化农业产业结构。加大治水改土力度，完成中低产田地改造30.8万亩。退出低产低质低效作物50万亩，改造中低产茶园12.6万亩，改造低产蔗园10.5万亩。新增高原特色农业产业化基地150.2万亩，其中，新植咖啡27.5万亩、澳洲坚果24.4万亩、核桃28万亩、竹木56万亩，其他作物14.3万亩。畜牧业产值达58.4亿元，增长8.3%；渔业产值达4.4亿元，增长14.1%。临沧华叶庄园基本建成，沧源碧丽源茶叶庄园、双江荣康达乌龙茶庄园、云县幸福农业循环经济庄园等一批农业庄园加快建设。临沧市“神十”龙胆草航天育种工程、云南航天生物科技实验示范基地启动建设。引导农民自愿、平等、有偿流转土地，构建新型农业经营体系，推进农村合作经济组织管理机制创新。全市新增规模以上农业龙头企业5户，新创建各类合作社290个，农业产业化组织总数达615个。

　　（二）工业发展步伐进一步加快。实施工业强市战略，推进二产发展绿色化、园区化。克服煤电油运资金等要素供给困难，加强企业服务工作，帮助企业正常生产，努力实现工业“双百”目标。着力构建以园区为载体的新型工业化发展格局，临沧工业园区规划面积扩大到50平方公里，10平方公里的新区建设项目开工。工业园区管理制度进一步完善，10大园区水、电、路、标准厂房等基础设施及配套建设完成投资40.6亿元，新增入园企业67户。全市共实施工业项目283个，一批工业项目竣工投产，一批在建项目快速推进，一批新建项目顺利开工。完成工业投资111.7亿元，增长22.3%，规模以上工业增加值100.2亿元，增长19.6%。加大扶持小型微型企业发展的力度，民营经济总量达7.2万户，增长16.8%，实现非公经济增加值166.7亿元，增长19.7%。

　　（三）第三产业提速发展。按照三产发展特色化、高端化要求，围绕“全域景区化”的思路，加快“大旅游”产业发展。完善旅游产业发展规划，出台旅游产业发展政策，打造旅游产业品牌。临沧被列入全国低碳国土实验区，获得“中国十佳绿色城市”、“中国恒春之都”荣誉称号，入选“2013年中国避暑避寒旅游城市”，凤庆古墨、沧源翁丁荣登“2013中国避暑小镇”排行榜，临翔南美、双江冰岛等12个村进入第二批中国传统村落名录，城市品牌、城市形象的影响力、知名度不断提高。“沧源国际旅游度假区”创建工作顺利启动。成功举办中国名村走进“世界佤乡·天下茶仓”活动暨全国“村长”论坛等系列活动，办好“中国沧源佤族司岗里摸你黑狂欢节”等民族文化节庆。临沧机场航班增加到每天4个，开通临沧经停昆明至北京、上海、成都、西安航线。接待国内外旅客461.6万人次，实现旅游收入30.8亿元，增长31.3%。临沧被确定为“亚洲微电影艺术节永久举办地”，成功举办首届亚洲微电影艺术节暨“金海棠”奖颁奖活动，亚洲微电影博物馆、产品交易中心一期工程建成投入运行。临沧中国网库云南生物产业电子商务基地项目20个单品交易平台落地并加快建设，中国红茶交易网等7个单品交易平台上线运营。邮电通讯、住宿餐饮、物流仓储、咨询信息等服务业加快发展，一批企业上规模、上档次。第三产业增加值达110亿元，增长12.9%。

　　（四）基础设施进一步改善。努力改变基础设施滞后状况，加快综合交通、水利、能源、信息等基础设施建设。祥云至临沧铁路前期工作扎实开展；玉溪新平至临沧临翔高速公路前期工作加快推进，羊头岩至振兴桥二级公路、国道214线双江过境公路建成通车，临翔至博尚、耿马至孟定快捷通道启动规划建设，云凤一级公路、沿边二级公路等项目加快建设，完成643公里建制村通畅工程；沧源得龙机场开工建设，临沧机场改扩建工程基本完成；澜沧江水运基础设施建设扎实推进。3件中型、3件小（一）型水库开工建设，2件烟草水源工程启动建设，在建的8件中型、11件小（一）型重点水源工程加快建设，南汀河孟定城区段河道治理工程顺利推进，完成山区“五小水利”工程5.5万件。解决了19.8万农村人口饮水安全问题。地方水电、农网改造等能源项目建设加快。顺利实施无线电基础设施建设工程，第三代移动通信网络覆盖面进一步扩大，第四代移动通信工程加快推进。

　　（五）人居环境进一步提升。围绕建设“微笑之城、创业之城、森林之城、洁净之城”四位一体美丽边城目标，进一步完善城镇建设规划，加快推进一批城市综合体、市政基础设施等项目建设。完成旧城改造102万平方米，城镇建成区面积增加4.4平方公里，城镇承载力进一步增强。续建的52296套保障性住房加快建设，新建的18630套保障性住房全面开工，3900户城镇低收入家庭搬入保障房。改善性商品住房建设顺利启动。8县（区）城市污水处理设施稳定运行，“两污”治理取得新进展，城市环境不断改善。合力建设“村美、民富、人欢乐”的美丽乡村，完成旧村（组）改造401个，农村危房改造4万户。双江、镇康分别获得“2013中国绿色竞争力十强县”和“云南省园林县城”称号。积极稳妥推进户籍制度改革，新增22.5万农业人口转变为城镇居民。

　　（六）生态文明建设强势推进。致力于实现“森林繁茂、水系发达、空气清爽、人文荟萃、经济循环”的生态文明建设目标，认真实施《临沧生态市建设规划》，加快森林临沧建设。云南南滚河、永德大雪山、临翔五老山、双江大浪坝等自然保护区、森林公园的保护与建设加快推进。加强林业有害生物防治和森林防火工作，森林资源得到有效保护。积极开展植树造林活动，完成造林139.2万亩。着力推进“十百千万”工程，共种植树木523.1万株。临沧城市森林公园和临翔博尚镇腾龙组、双江勐库镇忙蚌组森林村庄示范点建设等工程加快实施。全市森林覆盖率达65.2%。临翔花果山城市森林公园和凤庆安石村被评为省级生态文明教育基地。启动南汀河流域综合治理工程，关闭一批采石采砂场，实施一批退耕还林、陡坡地生态治理等工程，着力打造南汀河流域苗木产业带、生态景观带和林果产品交易带，生态综合治理取得明显成效。生态补偿、环境污染治理等制度有效落实，项目建设生态环境监管“三同时”执行率均达100%，34个省级重点减排项目加快实施，主要污染物排放明显减少，无较大环境污染事件发生。

　　（七）沿边开放水平进一步提高。紧紧抓住国家建设桥头堡战略机遇，加快推进沿边开发开放。临沧边合区获国务院批准成立，临沧在国家桥头堡战略中的地位和作用更加凸显，一批基础设施、产业、民生项目建设加快推进。成功举办第三届边境经济贸易交易会。缅北农业合作开发取得新进展。一批缅商入临投资城镇建设、农业庄园、农产品开发等领域。清登公路建设加快。边合区共实施项目219个，完成招商引资实际到位资金167.9亿元，实现规模以上固定资产投资105.7亿元，规模以上工业增加值15.8亿元，外贸进出口总额31.8亿元。

　　（八）财税金融运行平稳。把增强投融资能力作为推动各项工作的重要抓手，依法加强税收征管，大力发展实体经济，财源建设不断加强。积极推进“营改增”试点改革，落实财政预决算公开制度，财政资金管理进一步加强。优化财政支出结构，实现财政收支“双增”，地方公共财政预算支出完成181.3亿元。金融支持经济发展作用增强，各项人民币存款余额404.8亿元，增长16.6%；贷款余额321.1亿元，增长19.3%。新增7户小额贷款公司。商业保险健康发展，及时理赔甘蔗、烤烟、能繁母猪等农业保险。投资软环境进一步优化，荣获“2013浙商最佳投资城市”称号，成功举办“绿色之约·创富临沧-百家浙商临沧行”、省工商联十一届四次常委会议暨“民企携手佤山·成就大美临沧”等活动。招商引资国内合作项目达934个，实际到位资金510亿元，增长97%，其中，引进省外到位资金370.4亿元，增长131.4%；利用外资4622万美元，增长12.6%。

　　（九）城乡民生进一步改善。扎实推进教育、文化、科技、医药卫生体制改革，公共服务体系逐步健全。教育工作取得新突破，普通高考文理综合一本上线率排名全省第三，学生营养改善计划实施范围扩大到城区义务教育阶段农村学生。加快实施文化惠民工程，文化事业有新进步。加强医疗卫生服务能力建设，城乡居民健康水平进一步提高。科技惠民工程扎实推进，国家可持续发展实验区建设进一步加强。城镇新增就业1.5万人，新增农村劳动力转移就业20万人。发放“贷免扶补”、小额担保贷款等3.2亿元。城镇职工基本养老保险参保9.9万人，城乡居民社会养老保险参保127.9万人。参加城镇基本医疗保险22.9万人、失业保险8.3万人、工伤保险11.6万人、生育保险10万人。企业退休人员养老金每月人均增加212元。居民医保财政补贴标准由240元提高到280元。全面推行城镇居民医疗保险门诊统筹，职工大病补充保险限额由16万元提高到20万元。城镇20种重特大疾病住院费用报销比例提高到90%。新农合参合率达97.1%，农村23类大病纳入重大疾病保障试点范围。高度重视移民工作，移民扶持和稳定工作成效明显。加大少数民族和民族地区发展扶持力度，扶贫开发等工程建设加快，减少贫困人口10万人。切实做好防灾减灾工作，妥善安排好受灾群众的生产生活。驻临军警部队现代化建设、国防动员和民兵预备役工作取得新成效。完善信访和调解联动工作体系，预防和化解社会矛盾，无重大群体性事件发生。加大普法宣传教育和依法治市力度，保持严打高压态势，禁毒防艾、禁赌取得明显成效。社会创新和治理不断加强，“平安临沧”建设深入推进。

　　（十）政府自身建设进一步加强。深入开展“四群”教育和干部直接挂钩联系群众活动、党的群众路线教育实践活动、市情民情大调研、优化软环境增强软实力活动和“作风转变年、项目落地年、服务基层年”等活动。全面贯彻中央、省委、市委关于廉洁从政的规定，加大廉政建设和反腐败工作力度。切实发挥审计、监察职能作用，民生资金电子网络监察管理系统建设成效明显。坚决纠正和解决“四风”问题，厉行节约，反对浪费，“三公”经费下降10%。切实转变文风、会风，发文减少13.5%，会议精简12%。坚持重大行政审批备案、公示、听证制度，行政审批制度进一步完善。加强公共资源的监督和管理，推动公共资源交易“应进必进”。政务服务中心体系作用充分发挥，群众办事更加方便。自觉接受市人大及其常委会的法律监督和工作监督，执行市人大及其常委会的决定决议。支持市政协履行政治协商、民主监督、参政议政职能。积极支持人大代表、政协委员开展视察和调研工作。市人大代表批评、建议和意见以及市政协委员提案办复率均达100%。

　　各位代表，本届政府履职的五年，是改革发展任务十分艰巨繁重的五年。五年来，在省委、省政府和市委的坚强领导下，市人民政府团结和依靠全市各族干部群众，超额完成了五年工作的目标任务，“十二五”经济社会发展主要预期目标已提前完成。

　　——过去五年，综合经济实力明显增强。我们抢抓发展机遇，奋力推进经济持续快速发展，全市综合经济实力显著提升。生产总值从159亿元增加到416.1亿元，增1.6倍。地方公共财政预算收入从8.5亿元增加到36.9亿元，增3.3倍。规模以上固定资产投资从57亿元增加到417.3亿元，增6.3倍。城镇居民人均可支配收入从10274元增加到18563元，增长80.7%。农村居民人均纯收入从2363元增加到6066元，增1.6倍。消费品零售总额从48.5亿元增加到116.3亿元，增1.4倍。外贸进出口总额从6.5亿元增加到35.4亿元，增4.4倍。

　　——过去五年，产业结构明显优化。经济发展加快转型，三次产业结构由36.3:31.3:32.4调整为31.3:42.2:26.5。农村各项改革顺利推进，集体林权主体改革、农垦主体改革全面完成。高原特色农业加快发展，农业产业化基地由1076万亩增加到1802万亩，增加726万亩，增长67.5%。粮食生产实现“十连增”，总产量从74.9万吨增加到98.6万吨，增长31.6%。烤烟收购总量从26.9万担增加到110.2万担，增3.1倍，迈入全国大型烟区行列。农业增加值从57.7亿元增加到130.4亿元，增1.3倍。全市工业园区总体规划全面完成，4个园区已纳入省级园区规划加快培育，实现县域园区“一县一园”全覆盖。规模以上企业达96户，一批企业加快建立现代企业制度，云南锗业成功上市，龙润集团上市公司成功落户临沧。质量兴市取得新成效，3件商标获全国驰名商标认定，49件商标获云南省著名商标认定，18个产品获云南省名牌产品认定，5户企业获国家高新技术企业认定，11户企业获省级创新型试点企业认定。淘汰落后水泥生产能力55万吨、铁合金生产能力3600千伏安，38户工业企业通过清洁生产审核验收。旅游总收入从10.2亿元增加到30.8亿元，增2倍。第三产业增加值从51.6亿元增加到110亿元，增1.1倍。

　　——过去五年，基础设施明显改善。进一步完善政府投资项目决策机制，重大基础设施建设项目扎实推进。二级公路通车里程由436公里增加到1032.4公里，实现境内3个国家级口岸和县县通二级公路，形成全市互联互通的4小时经济圈。完成1914公里通畅工程、6411公里通达工程，乡村公路通畅率、通达率均达100%，在全省率先实现乡乡建有客运站。澜沧江航道里程达289.6公里，水运能力逐步增强。新开工建设11件中型、16件重点小（一）型水源工程，完成1件中型、46件小（一）型、134件小（二）型水库除险加固，建成10.7万件“五小水利”工程。解决了91.4万农村人口饮水安全问题，有效灌溉率提高到37.4%。完成中低产田地改造114万亩。地方水电站总装机达69.2万千瓦。农村人口用电覆盖率达100%。

　　——过去五年，生态环境明显改善。不断深化生态文明体制改革，加强生态环境建设，推进可持续发展。扎实开展“七彩云南·秘境临沧”保护行动，重点生态功能区保护全面加强。古茶古树、珍稀名木挂牌保护制度有效落实。境内分布的原始生态系统类型和90%以上的野生动植物物种得到有效保护。连续5年无重特大森林火灾。完成造林617.2万亩。完成退耕还林131.4万亩，陡坡地生态治理14万亩。完成水土流失治理1075.4平方公里，石漠化综合治理23.2万亩。森林覆盖率由46.6%提高到65.2%。8县（区）城市和重点城镇环境空气质量优良率为100%，水环境质量继续改善，集中饮用水源地水质达国家二级以上保护区要求。全市生态环境总体保持优良水平，在全国范围内的环境竞争力显著提升。

　　——过去五年，城镇化进程明显加快。按照“守住红线、统筹城乡、城镇上山、农民进城”的要求，调整土地利用、城乡建设、林地保护规划，严格保护耕地，土地综合利用率不断提高。临沧中心城市缅宁大道、玉龙湖、南汀河临沧城段治理等一批城镇项目竣工投入使用。开工建设城镇保障性安居工程76922套，竣工26000套，入住16000套。旧城改造439万平方米。城镇建成区面积从67.5平方公里增加到89.9平方公里。建成城市生活垃圾处理厂6座、污水处理厂8座。城市管理标准化和数字化全面推行。城市建成区绿化覆盖率达36%。完成“农转城”38万人，城镇化率从28%提高到33.9%。坚持城乡建设“双轮”驱动，全面完成8122户农村危房改造“佤山幸福工程”，全市改造旧村1603个村（组），改造危房23.3万户。城乡居民的生活环境进一步改善。

　　——过去五年，沿边开放明显加快。抢抓桥头堡建设机遇，构建开放型经济体制，以边合区建设引领沿边开发开放。编制完成《临沧市加快建设面向西南开放重要桥头堡总体发展规划》、《孟定城市总体规划》等一批边合区建设规划，一批基础设施、产业项目、民生项目扎实推进，对缅经贸、教育、文化、卫生等交流合作全面开展，合作开发境外特色农业产业基地111.8万亩。一口岸多通道、跨境旅游以及人民币跨境结算等申报工作进展顺利。成功承办云南省沿边开放工作现场会，连续举办三届临沧边境经济贸易交易会。边合区累计实施项目354个，实现招商引资实际到位资金249.3亿元，完成规模以上固定资产投资157.9亿元，外贸进出口总额52.4亿元，实现规模以上工业增加值25.3亿元。边合区逐步成为拉动全市经济发展的重要引擎。

　　——过去五年，财税金融工作不断加强。多措并举，抓好投融资工作。财税体制改革、云县“扩权强县”试点等改革不断深化。争取中央、省资金506亿元。财政总收入跃上60亿元台阶，公共财政预算支出从49.2亿元增加到181.3亿元，增2.7倍。投融资体制改革取得初步成效，新搭建城市投资公司、水务投资公司、工业投资公司、林业投资公司、临沧边境经济合作区投资开发公司等市级融资平台。金融体系建设逐步加强，上海农商行入驻临沧，组建19户小额贷款公司、5户融资担保公司。与省级银行金融机构开展合作，存款余额从134.9亿元增加到404.8亿元，增2倍；贷款余额从99.7亿元增加到321.1亿元，增2.2倍。成功发行首支5亿元企业债券。招商引资成果丰硕，国内合作项目从171个增加到934个，增4.5倍；实际到位资金从33.9亿元增加到510亿元，增14倍。财税金融为经济社会发展提供了强有力的支撑和保障。

　　——过去五年，城乡民生普遍改善。切实把保障和改善民生作为工作的根本出发点和落脚点，财政投入民生的支出占70%以上。教育领域综合改革顺利推进，基本实现“两基”目标。义务教育阶段农村学生营养改善计划实现城乡全覆盖。在全省率先完成中小学D级危房拆除重建任务，建成标准化中小学97所。职业教育加快发展，临沧师专专升本工作扎实推进。医药卫生体制改革稳步推进，4个县级公立医院综合改革试点启动实施。建成标准化乡镇卫生院56个，标准化村卫生室464个。全面完成人口与计生工作目标任务，妇女儿童保健水平逐步提高。文化体制改革扎实推进，文化惠民工程加快实施。广播电视实现村村通，广播电视覆盖率达98%。深入推进国家可持续发展实验区建设，科技进步对经济增长贡献率达49%。城镇新增就业61782人，开发公益性岗位7193个。养老、医疗、失业、工伤、生育“五项保险”实现全覆盖，保障水平进一步提高。连续5年提高企业退休人员养老金。新农合参合人数达194.6万人。社会救助惠及224万人（次），184万人（次）灾民生产生活得到有效安置。“兴边富民”、扶持人口较少民族发展和边疆解“五难”工程扎实推进。减少贫困人口51.8万人。电站库区等工程性移民安置和后期扶持工作加快推进。认真贯彻党的民族宗教政策，民族团结进步边疆繁荣稳定示范区建设扎实推进，民族团结进步和谐。全力支持驻临军警部队现代化建设、国防动员和民兵预备役建设，双拥工作深入开展，连续两届荣获“全国双拥模范城”称号，军政、军民关系更加和谐。食品药品安全和安全生产形势总体稳定，普法和依法治市工作扎实推进，社会治安综合治理全面加强。禁毒防艾人民战争深入推进。严厉打击涉黄、涉毒、涉枪、涉拐和跨境赌博等违法犯罪活动取得明显成效。全市民族团结、边疆稳定、社会和谐的良好局面进一步巩固。

　　——过去五年，政府自身建设进一步加强。深入推进法治政府、责任政府、阳光政府和效能政府建设。政府机构改革、事业单位分类改革顺利推进，行政审批制度改革有效实施，市级审批事项由原来的424项精简到63项，成为全省审批事项最少的州市之一。市、县、乡、村四级政务服务中心体系基本建成。深入开展学习实践科学发展观活动、向杨善洲同志学习活动、“创先争优”活动、“四群”教育和干部直接挂钩联系群众活动、党的群众路线教育实践活动、优化软环境增强软实力活动、市情民情大调研等活动，党群、干群关系进一步密切。自觉接受市人大及其常委会的法律监督和工作监督，自觉接受市政协的民主监督，办理市人大代表批评、建议和意见393件，办复率100%；办理市政协委员提案601件，办复率100%。广泛听取民主党派、工商联、无党派人士的意见和建议，保障新闻媒体、各界人士的知情权、参与权、表达权、监督权。政府依法行政能力和社会公信力不断增强。

　　五年来，中国特色社会主义理论体系深入宣传，群众性精神文明创建活动广泛开展，积极支持关工委、妇儿工委、工会、共青团和妇联开展工作，群团组织参与社会管理和公共服务的桥梁纽带作用充分发挥。“七彩云南全民健身工程” 深入实施。外事、侨务、对台等工作扎实推进。工商、质监、粮食、供销、档案、地方志工作取得新成绩。科协、老龄、慈善、残疾人事业持续推进。红十字会作用充分发挥。文联、社科联服务经济社会发展取得新进展。审计、统计调查的信息、咨询、监督职能充分发挥。人防工程专项整治取得实效。防震、气象、水文服务能力进一步提高。

　　回顾第二届市人民政府的工作，我们深深体会到，要加快临沧经济社会发展，必须做到“七个坚持”。

　　——坚持以科学发展观为指导，坚定不移走“生态立市，绿色崛起”之路，稳中求进，开拓创新，努力建设天地人高度和谐的大美临沧，谱写好“中国梦”临沧篇章。

　　——坚持改革开放，突出经济体制改革重点，加快破除经济、政治、社会、文化和生态建设的体制机制障碍，彰显沿边区位优势，努力提高沿边开发开放的层次和水平。

　　——坚持打牢发展基础不动摇，全力破解交通瓶颈制约，全面推进水利、能源、信息等基础设施建设，努力增强基础设施对经济社会发展的支持作用。

　　——坚持统筹城乡发展，转变发展方式，实行以城带乡、以工促农、城乡互动、协调发展，形成发展新格局和竞争新优势，逐步缩小城乡发展差距。

　　——坚持科教与人才兴市战略，充分发挥科技教育在经济社会发展中的重要支撑作用，优先发展教育，全面实施素质教育，促进教育全面协调发展，培养与引进相结合，加快现代化人才队伍建设，推进创新型临沧建设。

　　——坚持产业兴市，大力发展实体经济，加快培育高原特色农业，强势推进绿色工业，着力发展现代服务业，做强做大支柱产业，努力构建区域特色产业体系，增强产业市场竞争力。

　　——坚持保障和改善民生，走各民族团结进步、共同繁荣之路，争当推进民族团结进步事业的排头兵。加快发展社会事业和基本公共服务，促进社会公平正义，加强和创新社会治理，保持社会和谐稳定。

　　各位代表！五年来取得的成绩来之不易，积累的经验弥足珍贵。这是省委、省政府和市委正确领导、科学决策的结果，是全市各族人民团结奋斗、开拓进取的结果。在此，我谨代表市人民政府，向广大工人、农民、干部、知识分子，向人大代表、政协委员、各民主党派、工商联和无党派人士、各人民团体、社会各界人士，向驻临人民解放军和武警部队官兵，向所有关心支持临沧发展的港澳同胞、台湾同胞、海外侨胞和国际友好人士，表示崇高的敬意和衷心的感谢！

　　在充分肯定成绩的同时，我们也清醒地认识到，临沧仍然是欠发达的边疆民族地区，加快发展面临着诸多困难和问题：深化改革任务繁重，维护稳定责任重大；基础设施建设滞后，交通瓶颈制约突出；经济总量小，人才支撑不足，增长方式粗放，调结构转方式任务艰巨；城乡居民增收难度大；财政收支矛盾突出；思想解放不够，工作水平和效率亟待提高，作风建设还需加强。针对这些困难和问题，我们必须采取更加有力的措施，认真加以解决，确保经济社会取得更大发展。

　　二、今后五年的目标任务

　　未来五年，我们迎来了加快发展的机遇：一是随着全面深化改革的力度进一步加大，必将激发经济社会发展的内生动力与活力。二是国家高度重视西部大开发和沿边开放，特别是临沧边境经济合作区获国务院批准，纳入国家沿边金融综合改革试验区范围，国家、省对西部沿边地区扶持力度加大。三是东部地区产业向西部地区转移进程加快，给我市承接产业转移、加快结构调整创造良好的条件。四是我市“生态立市，绿色崛起”发展理念蕴藏着广阔的发展空间和巨大的发展潜力。五是近年来我市建成了一批重大基础设施和重要产业项目，为加快发展创造了条件。我们要抢抓机遇，乘势而上，努力实现经济社会发展跃上新台阶。

　　今后五年政府工作的总体思路是：高举中国特色社会主义伟大旗帜，坚持以邓小平理论、“三个代表”重要思想、科学发展观为指导，深入贯彻落实习近平总书记系列重要讲话精神，按照党的十八大，十八届二中、三中全会，省委九届七次全会和市委三届五次全会的部署，牢固树立“生态立市，绿色崛起”的发展理念，以“构筑生态高地，成就大美临沧”为目标，把改革创新贯穿于经济社会发展的各个领域各个环节，坚持稳中求进工作总基调，做到稳中有快、稳中提质、稳中增效，在全面实现“十二五”工作目标任务的基础上，确保今后五年经济社会发展实现新跨越。

　　今后五年全市经济社会发展主要预期目标建议为：生产总值年均增长12%以上，地方公共财政预算收入年均增长12%以上，城镇居民人均可支配收入年均增长12%以上，农村居民人均纯收入年均增长14%以上，城镇化率和森林覆盖率进一步提高。全市经济发展、社会进步、文化繁荣、民族团结、民生改善、生态优良、边境稳定的大好局面更加巩固。

　　在今后五年的工作实践中，我们必须以改革发展绿色崛起统领全局，突出抓好以下工作：

　　——全面深化各项改革。坚决贯彻落实党中央、国务院，省委、省政府和市委关于深化改革的决策部署，坚持正确、准确、有序、协调推进各项改革。突出抓好生态文明建设、美丽家园建设和边境经济合作区建设相关的各项改革。重点推进农村综合改革、经济体制改革、现代企业制度改革、投融资体制改革、财税体制改革、沿边金融综合改革、资源性产品价格改革。继续深化行政审批制度改革、教育领域综合改革、医药卫生体制改革、科技创新体制改革、文化体制改革、社会治理体制改革。让改革发展成果更多更公平更直接地惠及全市各族人民。

　　——加快调整产业结构。加快科技创新，努力培育绿色低碳产业，推动三次产业融合发展。突出现代农业示范区、产业园和农业庄园建设，加快提升传统农业，大力发展高原特色农业。突出工业园区建设，积极发展装备制造、清洁能源、新材料、农产品林产品加工业、轻纺工业，推进产业集约集群集聚发展，加快构建绿色工业体系。大力发展总部经济、飞地经济、庄园经济。加快发展生态文化旅游、商贸物流、金融保险、休闲度假、养生养老、电子商务、文化产业等现代服务业。

　　——全力改善基础设施条件。加快构建综合交通体系，力争祥云至临沧铁路、玉溪新平至临沧临翔高速公路建成通车，确保沿边二级公路建成投入使用，沧源得龙机场、临沧港至缅甸航运通道建成通航。加快构建现代水利系统，实施一批重点水源工程和“五小水利”工程，力争有效灌溉率达54%。继续加强能源、信息化网络等基础设施建设。实现基础设施滞后状况明显改变，为加快发展创造更好的条件。

　　——切实加强生态文明建设。全面加快森林临沧建设，划定严格的生态保护红线，扩大森林、湿地等绿色生态空间比重。突出抓好南汀河一线、边境一线、澜沧江一线、城镇沿河面山等重点区域生态综合治理。严格实行古茶古树、珍稀名木挂牌保护制度。大力发展森林文化、茶文化、竹文化。加快建设高山水系，建设发达的现代水循环系统，打造好生态环境品牌。

　　——加快沿边开发开放。加快推进桥头堡临沧前沿窗口建设，主动对接国家建设丝绸之路经济带、21世纪海上丝绸之路“一带一路”重大战略，积极融入孟中印缅经济走廊和缅甸皎漂、密支那经济合作区建设，探索对内对外合作新模式，构建开放型经济新体系。搭建内联外通的陆路网络，构建边境特色产业体系，加强边境城镇和口岸建设，提高通关便利化水平，实现与缅甸以及南亚、东南亚国家向更宽领域更深层次合作。全力把边合区建设成为全市经济发展重要增长极。

　　——认真做好投融资工作。紧紧抓住深化财税金融投资体制改革的机遇，积极争取中央、省转移支付。注重财源建设与结构调整相结合，全面提高财源建设质量。加大依法治税力度，不断提高税收征管水平。强化财政收支的管理，提高资金的使用效益。加强金融体系建设，增强金融对经济社会发展的支持能力。优化招商引资环境，不断创新招商引资方式，拓展招商引资领域，努力实现招商引资“质”和“量”的新突破。

　　——持续改善城乡民生。全面发展教育、卫生、科技、文化等各项社会事业，促进公共服务均等化。鼓励创业、扶持就业，不断提高就业水平。认真落实城乡养老保险、医疗保险等社会保障制度。落实好城乡低保政策，加快社会救助体系建设，有效救助弱势群体。加强监管，保障食品药品安全。向贫困宣战，抓好集中连片特困地区扶贫开发，搞好精准扶贫，不断减少贫困人口。努力实现“学有所教、病有所医、住有所居、劳有所得、老有所养、困有所助”。

　　三、2014年的重点工作

　　2014年是全面深化改革，加快绿色崛起的关键之年。我们要认真贯彻党中央、国务院，省委、省政府和市委的重大决策部署，坚持“生态立市，绿色崛起”的发展理念，以“构筑生态高地，成就大美临沧”为目标，着力抓好20项重要工作（见附件1）和“四个一百”上亿元重大项目建设，全面完成42项重要指标，抓改革促发展、抓创新树品牌、抓投资稳增长、抓产业强支撑、抓基础破瓶颈、抓城镇增活力、抓开放扩空间、抓生态保持续、抓民生促和谐，确保全市经济持续健康发展、社会和谐稳定。　　2014年经济社会发展的主要预期目标建议为：

　　——地区生产总值增长12%以上；

　　——规模以上固定资产投资增长28%以上；

　　——地方公共财政预算收入增长12%以上；

　　——城镇居民人均可支配收入增长12%以上；

　　——农村居民人均纯收入增长14 %以上；

　　——社会消费品零售总额增长13%以上；

　　——外贸进出口总额增长15%以上；

　　——人口自然增长率控制在6‰以内；

　　——城镇登记失业率控制在4.3%以内；

　　——居民消费价格总水平涨幅控制在3.5%左右；

　　——单位生产总值能耗下降0.8%。

　　为实现上述目标，今年市政府将着力抓好以下十一个方面的工作：

　　（一）积极推进各项改革，努力破解体制机制障碍。继续深化农村改革，加快推进农村土地确权登记颁证工作，推动农村承包土地和林地使用权适度流转，推进集体林权制度配套改革，有序开展农村土地承包经营权证、房屋所有权证和林权证“三权三证”抵押融资工作。深化生态文明体制改革，严格实施主体功能区规划，建立生态红线管控制度，健全自然保护区、森林公园等重点生态功能区管理制度，落实生态补偿政策。深化财税体制改革，推进政府和部门预决算公开，扩大“营改增”行业范围，认真落实结构性减税政策。深化资源性产品价格改革，加快推进农村水利、饮水项目管理体制和农业综合水价改革，抓好涉及民生的价格和收费管理。健全城乡规划建设管理体制，加强城市空间开发利用管制。抓好户籍制度改革，促进农业人口就地城镇化。继续推进供销社改革、沿边金融综合改革。全面深化教育领域综合改革，促进教育均衡发展。深化医药卫生体制改革，完善基层医疗卫生机构运行新机制，加快县级公立医院改革。稳步推进新一轮政府机构改革和事业单位分类改革，深化行政管理体制改革和行政审批制度改革，进一步精简行政审批事项。推进社会组织管理制度改革，鼓励和支持社会力量参与社会治理、公共服务。全面完成中央、省委、市委部署的改革任务。

　　（二）大力发展特色农业，打牢农民增收基础。围绕“农业强、农村美、农民富”目标，加快推进高原特色农业发展。守住耕地红线。完成治理水土流失265平方公里，中低产田地改造25万亩。全面落实粮食行政首长负责制，确保粮食总产量达101万吨。加快推进南汀河流域10个生态农业园和临翔至双江5个现代农业产业园建设。退出低产低质低效作物50万亩，新发展产业化基地150万亩以上，其中，新增咖啡10万亩、竹木65万亩，澳洲坚果、核桃等木本油料60万亩。建设生态茶园5万亩。建成“万元田”10万亩、“万元山”10万亩，培育“万元人”10万人。积极发展庄园经济，重点建设40个农业庄园。实现甘蔗产量645万吨，茶叶产量10万吨，烤烟产量102.2万担；实现畜牧业产值60亿元，渔业综合产值10亿元；加快培育新型经营主体，新增农业产业化组织100个，新增农业龙头企业10户。实现农业增加值150亿元，增长6%以上。

　　（三）突出工业园区建设，加快发展绿色工业。认真实施工业强市战略，按照“绿色、低碳、节能、环保、循环”要求，继续推进糖业二次创业，振兴茶产业、酒及饮料产业，发展烤烟、咖啡、坚果、生物药业、木竹等绿色农产品加工业，加快发展以水电、太阳能、风能、生物质能等为主的清洁能源产业，以锗、硅系列产品开发和装备制造、生物医药为主的新兴产业。加快临沧工业园区东扩新区建设，扎实推进凤庆滇红园区、云县光伏园区等工业园区建设，规划建设临沧华侨创业园。力争工业园区固定资产投资达90亿元，努力实现全市工业园区产值占工业总产值60%以上。新开工55个项目，加快建设52个项目，力争年内竣工80个项目。实现工业规模以上固定资产投资132亿元，增长20%。增强工业要素保障，确保企业正常生产。实现全社会用电量达26亿千瓦时。促进信息化与工业化深度融合，支持企业自主创新，增强工业竞争力。实施中小企业成长工程，重点扶持21户重点工业企业（见附件2），再引进一批民企入临发展。实现规模以上工业增加值120亿元，增长20%，实现民营经济增加值200亿元以上，占全市生产总值40%以上，企业从业人员达23万人以上。推行清洁生产，完成节能减排目标任务。

　　（四）加快培育现代服务业，推进第三产业提速发展。巩固提升传统服务业，加快发展现代服务业。注重发展养老服务业和健康休闲业，满足人民群众日益增长的健康服务需求。以建设沧源国际旅游度假区为重点，推进临沧全域景区化建设。有效整合特色旅游资源要素，强化景区景点、旅游设施等基础设施建设，加快旅游综合体建设，规划建设佤文化旅游精品景区、文化主题园区。加快建设5个特色旅游小镇，推进10个避暑避寒恒春小镇创建工作。规划建设30个特色旅游村，集中打造一批精品旅游农庄和乡村庭院。办好“摸你黑”等节庆活动。举全市之力办好第二届亚洲微电影艺术节，确保亚洲微电影网正常运行，推动文化产业加快发展。加快建设临沧中国网库云南生物产业电子商务基地项目20个单品交易平台，推进电子商务加快发展。不断完善城乡市场流通体系，加快推进一批重大商贸流通、乡镇集贸市场、农副产品专业市场等项目建设。

　　（五）加强城乡建设，努力提升城乡人居环境。按照“以人为本、规划优先，地域特色、国际表达，历史文化、未来延伸，特色建筑、经典实用，优越生态、共建共享”的原则，加快森林城乡建设，全力打造美丽边城。着力构建边境一线、凤庆-云县-临翔-双江一线和永德一点的“两线一点”城市格局。加快“临沧主城区一城两片一体化、耿孟一体化、云凤一体化”发展。持续推进城镇上山，打造特色城市，避免千城一面。高标准、高水平、高质量打造临翔中心城市和七县城、孟定镇。加快推进城镇“一水两污”设施和城市综合体建设。新开工建设4600套城镇保障性安居工程，加快推进改善性商品住房建设。统筹老城区、城市新区协调发展，完成100万平方米旧城改造。积极推进产城融合，将中心城区扩容提质与工业园区扩能增效结合起来。促进房地产业和建筑业健康发展，实现房地产增加值增长10%以上，建筑业增加值增长25%以上。启动凤庆、云县撤县设区，加快撤乡建镇（办事处）步伐。深化户籍制度改革，保障进城农民权益，完成“农转城”10万人，城镇化率达36%。加强城市管理，做好园林城市创建工作。广泛开展爱国卫生运动，积极创建国家卫生城市。重点建设100个美丽村庄，建设2个特色精品村，完成农村危房改造4万户。

　　（六）加强基础设施建设，不断改善发展条件。始终坚持重大项目带动，突出抓好20个重点项目建设（见附件3）。力争开工建设祥云至临沧铁路，加快推进玉溪新平至临沧临翔高速公路前期工作，加快推进临翔至博尚、耿马至孟定快捷通道建设，加快推进云县至凤庆一级公路、沿边二级公路建设，完成孟定清水河至登尼境外公路、省道319线临翔过境公路建设，完成续建通畅工程1060公里、续建县乡道改造94公里，争取新开工通畅工程1000公里以上和县乡道改造工程40公里，完成澜沧江小湾、糯扎渡库区水运基础设施在建项目。力争沧源得龙机场完成投资8亿元以上。年内完成3件中型水库项目建议书工作，加快推进11件在建中型水源工程、16件重点小（一）型水源工程，新开工5件重点小（一）型水库，实施“五小水利”工程5万件以上。有效灌溉率达41%以上，解决16万农村人口饮水安全问题。加快推进水电、电网主网及农村电网升级改造等能源项目建设。加快“智慧临沧”、“第四代通信网络”项目建设。

　　（七）推进生态文明建设，创造良好生产生活环境。围绕 “森林繁茂、水系发达、空气清爽、人文荟萃、经济循环”建设目标，加快生态文明建设，呵护我们的共同家园。加强自然保护区建设，促进生物多样性保护。坚持生物措施与工程措施并举，重点抓好南汀河流域生态综合治理，打造苗木产业带、生态景观带和林果产品交易带。确保南汀河孟定主城区段防洪工程竣工，新开工南汀河干流中上游段治理等项目，加快推进南汀河军赛勐简段治理、耿马坝引水工程等项目建设，有效改善南汀河流域生态环境和城乡生产生活条件。确保完成退耕还林及荒山造林100万亩、2000万株。继续推进“十百千万”工程，完成树木种植521万株，森林覆盖率达66%。注重环境保护，建立健全污染综合防治体系，加强重点行业、重点区域污染治理，保持空气质量，保障饮用水源地和农产品基地环境安全。

　　（八）着力边合区建设，全面推进沿边开发开放。全力争取并实施好省政府支持临沧边合区建设的若干政策，加快边合区城镇、基础设施、产业、民生项目建设，继续推进缅北农业合作开发，确保边合区招商引资到位资金200亿元以上，完成规模以上固定资产投资150亿元以上，实现规模以上工业增加值20亿元以上，完成进出口总额38亿元。努力推动人民币跨境结算，提高携带人民币现钞进出境限额，开展外汇特许兑换业务等工作。积极推进中缅双边及第三国人员持护照通行、口岸升格、一口岸多通道、设立海关特殊监管区申报工作，努力推进通关便利化。深化友城合作，加强与缅甸等国家和地区的经贸、旅游、教育、卫生、科技、文化、边境维稳等交流合作，宽领域纵深推进沿边开发开放。

　　（九）抓好财税金融工作，努力增强保障能力。积极争取中央和省资金支持，加大税源培植和税收征管工作力度，推进财政收支增长，优化支出结构，确保完成地方公共财政预算收入41亿元以上，地方公共财政预算支出203亿元以上。加大对民生和重点项目的保障力度，严格控制“三公经费”等一般性财政支出。重视引进市外金融机构，鼓励发展小额贷款公司、担保公司。深化银政企合作，鼓励银行业金融机构扩大信贷规模，确保市内金融机构新增融资80亿元。大力发展各类商业保险，积极实施政策性保险。以办好中华茶商临沧行、第四届边交会等重大活动为契机，加大央企、省企、民企、外企入临力度。完成招商引资实际到位资金540亿元，其中：省外到位资金443亿元，利用外资6000万美元；新签约项目开工率达50%以上。

　　（十）推进各项社会事业发展，全力保障和改善民生。优先发展教育，逐步扩大普通高中和中等职业教育办学规模，支持发展民办教育、特殊教育，加快学前教育发展，推进义务教育均衡发展。加快滇西民族学院的建设和申报工作。继续实施农村义务教育阶段学生营养改善计划。新建义务教育标准化学校50所。深入实施“文化惠民工程”、“七彩云南全民健身工程”。启动茶叶原生地世界遗产申报工作，加快佤文化、茶文化、边地文化、休闲养生文化的开发与保护。全力筹办全国女子柔道锦标赛。加快建设国家可持续发展实验区，依靠科技增强可持续发展能力，提高科技进步对经济增长贡献率。狠抓卫生基础设施建设，新增住院病床900张。加强城乡公共卫生服务体系建设，强化疾病预防控制。坚持计划生育基本国策，抓好人口与计生工作，切实提高出生人口素质。落实积极就业政策，确保城镇新增就业2万人，增长30%，完成农村劳动力就业培训15万人，新增农村劳动力转移就业20万人。加快推行企业职工工资集体协商制度，严格执行最低工资标准，努力维护劳动关系和谐稳定。认真落实各项社会保险政策，提高参保率。全面推行城乡大病医疗保险，将新农合筹资标准提高到每人每年380元，新农合参合率达98%以上。新增养老床位1050张。加快助残服务体系建设。持续做好电站库区移民工作。加快实施扶贫攻坚工程，减少贫困人口12万人。办好10件惠民实事（见附件4）。加强和创新社会治理，建设市、县、乡三级视频接访系统，坚决维护群众利益。认真落实党的民族宗教政策，全力推进示范区建设，促进民族团结、宗教和谐。加强依法治市和普法工作，打好第三轮禁毒防艾人民战争。增强防恐意识，依法打击各类刑事犯罪和暴力恐怖犯罪活动。加强社会治安综合治理，维护社会稳定。高度关注边境动态，切实维护国家安全和边境安宁。加强双拥工作，支持驻临军警部队现代化建设、国防动员和民兵预备役建设，促进军民融合发展。严防安全生产事故发生，强化食品药品安全工作。加强工商、质监、物价工作，维护市场秩序。完成多普勒天气雷达系统建设，提高气象、水文服务能力。启动市人防疏散基地建设。继续支持工会、共青团、妇联、科协、工商联、社科联、关工委、妇儿工委做好工作。促进老龄、红十字、慈善、残疾人等事业健康发展。加强统计调查、供销、粮食、档案、地方志、文联、防震、外事、侨务、对台等工作。

　　（十一）加强政府自身建设，努力提高行政效能。按照建设法治政府、创新政府、廉洁政府的要求，进一步强化依法行政的理念，严格遵守党纪国法。深入开展党的群众路线教育实践活动，着力解决“四风”突出问题、群众反映强烈的切身利益问题和联系服务群众“最后一公里”的问题。严格执行党中央、省委、市委关于廉洁从政的若干规定，深入推进以完善惩治和预防腐败体系为重点的反腐倡廉建设，加强监察、审计等行政监督，强化对各级各部门重点环节、关键岗位行政权力的监督和制约，确保行政权力依法、公正、有效运行。建立健全决策咨询制度，完善重点工作和重大项目任务分解落实机制，认真落实行政问责、首问首办、服务承诺、限时办结等制度，全面提高行政效率。加快政务服务体系建设，简政放权，不断提高政务公开和政务服务规范化水平。建立健全政府购买服务机制，加大政府购买公共服务力度。自觉接受市人大及其常委会的法律监督和工作监督，执行市人大及其常委会的决议决定，自觉接受市政协的民主监督，积极采纳市政协的意见建议。认真办理人大代表批评、意见、建议和政协委员提案。虚心听取和采纳民主党派、工商联、无党派人士、各人民团体的意见，接受新闻舆论监督和社会公众监督，努力建设人民满意的服务型政府。

　　各位代表！新形势、新机遇、新任务催人奋进，让我们紧密团结在以习近平同志为核心的党中央周围，在市委的坚强领导下，进一步解放思想，坚定信心，团结奋进，努力拼搏，为建设天地人高度和谐的大美临沧，圆满实现今年经济社会发展目标任务而努力奋斗！
